# The Regulatory Role of Neuropeptide Gene Glucagon in Colorectal Cancer: A Comprehensive Bioinformatic Analysis

**DOI:** 10.1155/2022/4262600

**Published:** 2022-03-18

**Authors:** Wenfeng Du, Yue Miao, Guoqing Zhang, Guangcai Luo, Peng Yang, Fei Chen, Benhua Zhang, Chenggang Yang, Gang Li, Jin Chang

**Affiliations:** ^1^Department of Gastrointestinal Surgery, Liaocheng People's Hospital, No. 67 Dongchang West Road, Dongchangfu District, Liaocheng 252000, Shandong Province, China; ^2^Department of Radiation Oncology, The Second Affiliated Hospital of Shandong First Medical University, Taishan Street No. 366, Tai'an, 271000 Shandong Province, China; ^3^Department of Gastroenterology, The Second Affiliated Hospital of Shandong First Medical University, Taishan Street No. 366, Tai'an, 271000 Shandong Province, China; ^4^Department of Vascular Surgery, The Second Affiliated Hospital of Shandong First Medical University, Taishan Street No. 706, Taian 271000, China

## Abstract

**Background:**

Colorectal cancer is highly prevalent and causes high global mortality, and glucagon axis has been implicated in colon cancer. The present study is aimed at investigating the regulating mechanisms of glucagon involvement in colorectal cancer.

**Methods:**

Publicly available data from the TCGA database was utilized to explore the expression pattern and regulating role of glucagon (GCG) in colorectal cancer (COADREAD) including colon adenocarcinomas (COAD) and rectum adenocarcinomas (READ). Statistical analyses were performed using the R software packages and public web servers. The expression pattern and prognostic significance of GCG gene in pan-cancer and TCGA-COADREAD data were investigated by performing unpaired and paired sample analyses. The association of GCG expression with clinical characteristics was investigated using logistic regression analysis. Univariate cox regression analysis was performed to test the prognostic value of GCG expression for overall survival in COADREAD patients. GCG-significantly correlated genes were obtained. Biological functions and signaling pathways were identified by performing functional enrichment analysis and Gene Set Enrichment Analysis (GSEA). Additionally, the potential involvement of GCG in tumor immunity was researched by investigating the correlation between GCG expression and 24 tumor infiltrating immune cells.

**Results:**

GCG was found to be significantly downregulated in COADREAD tumor samples compared with healthy control samples. GCG gene was shown to be associated with the prognostic outcomes of COADREAD, whereby its upregulation predicted improved survival outcomes. Functional enrichment analysis showed that the top 100 positively and top 100 negatively GCG-correlated genes were mainly enriched in three signaling pathways including ribosome, nitrogen metabolism, and proximal tubule bicarbonate reclamation. The GSEA showed that GCG-significantly correlated genes were mainly enriched in cell cycle-related pathways (reactome cell cycle, reactome cell cycle mitotic, reactome cell cycle checkpoints, reactome M phase, Reactome G2 M DNA damage checkpoint, and Reactome G2 M checkpoints), neuropeptide ligand receptor interaction, RHO GTPases signaling, WNT signaling, RUNX1 signaling, NOTCH signaling, ESR signaling, HCMV infection, and oxidative stress-related signaling. GCG was positively correlated with Th17 cells, pDC, macrophages, TFH cells, iDC, Tem, B cells, dendritic cells, neutrophils, mast cells, and eosinophils and was negatively associated with NK cells.

**Conclusions:**

GCG dysregulation with high prognostic value in COADREAD was noted. Several tumor progression-related pathways and tumor immune-modulatory cells were linked to GCG expression in COADREAD. Therefore, GCG may be regarded as a potential therapeutic target for treating colorectal cancer.

## 1. Introduction

Colorectal cancer, which includes colon and rectal adenocarinomas (COADREAD), affecting the colon and rectal tissue, is among the top three cancers worldwide and the fourth most frequent cause of cancer-related death [1]. Furthermore, it is projected that in the coming decade colorectal cancer deaths will increase by more than 60%, owing to changing demographic trends and ageing populations [1]. The risk for colorectal cancer is largely ascribed to environmental factors including high dietary meat and fat intake with low fiber based diets, which alters the colonic microbiome and reduces the amount of anti-inflammatory short chain fatty acids such as butyrate [2]. Other established risk factors include smoking and alcohol use along with carcinogenic content in food or water, and it is purported that genetic differences may account for variable susceptibility to colorectal cancer [3].

The neuroendocrine peptide glucagon (GCG) is implicated in colorectal cancer [4–8]. Previous research has shown that aberrant GCG gene expression distinguished colorectal cancer tissue from hyperplastic polyps with 100% sensitivity [5]. Others have shown in a preclinical model that colon protective diets altered GCG transcript expression [6]. In human colon cancer cell lines, GCG activates its receptor, which leads to cancer cell proliferation by affecting AMPK/MAPK signaling [7]. In vivo, GCG infusion was shown to increase tumor-fractional protein synthesis in rectal cancer [8]. Glucagon gene is expressed in pancreas and L-type endocrine cells of the intestinal epithelium, where its processing leads to the formation of glicentin and GCG-like peptides 1 and 2 (GLP 1 and GLP 2) secreted in response to nutrient ingestion [9]. GLP-2 in particular is implicated in maintenance of the intestinal mucosal integrity and barrier function by its effects on epithelial permeability [10], indicating the potential for therapeutic targeting of GCG and GLP in colonic diseases. However, the role of GCG and GLP 1 and 2 in colorectal cancer is yet to be fully elucidated. In a preclinical model, chronic GLP 2 treatment was shown to promote colon carcinogenesis [11]. With regard to GLP 1, protective effects have been suggested in colon cancer [12], suggesting the role of recently developed GLP 1 agonistic drugs for diabetes mellitus [13] such as DPP-IV inhibitors in colorectal cancer.

Taken together, current evidence regarding the functional role of GCG and its associated proteins in COADREAD suggest significant gaps in knowledge in this domain. Comprehensive bioinformatics analysis of large, publicly available datasets has the potential to uncover relevant molecular mechanisms in this regard. Therefore, the present study was designed to leverage the publicly available TCGA database and perform a series of bioinformatics analysis to discover the putative role and functional mechanisms of GCG in COADREAD.

## 2. Materials and Methods

### 2.1. Expression of GCG in Pan-Cancer Data

The mRNA expression of GCG in pan-cancer data was analyzed and visualized by using the “ggplot2” package (version 3.3.3) in R program (version 3.6.3). RNAseq data from TCGA and GTEx in TPM (transcript per million) format was downloaded from UCSC XENA (URL: https://xenabrowser.net/datapages/), uniformly processed by the Toil process. Transcripts mapped data were normalized to TPM format and then log2 transformed. The XENA-TCGA data set consisted of 10,534 samples. The Mann–Whitney *U* test (also called the Mann–Whitney Wilcoxon Test or the Wilcoxon Rank Sum Test) was used for comparing differences in GCG mRNA expression between two groups (i.e., healthy control group and tumor group).

### 2.2. Expression of GCG in COADREAD Based on TCGA Data

Level 3 HT-seq data of COADREAD patients with the FPKM format were downloaded from the TCGA database. The samples without clinical information were removed. Thereby, 698 samples containing 647 COADREAD tumor samples and 51 adjacent healthy control samples were included for subsequent analysis based on TCGA-COADREAD data. The RNAseq data with FPKM (fragments per kilobase per million) format was normalized into TPM (transcripts per million reads) format and then log2 transformed. The mRNA expression of GCG gene in COADREAD was analyzed and visualized by using the ggplot package (version 3.3.3) in R program (version 3.6.3). Unpaired and paired sample analyses were both performed. For paired data satisfying the Shapiro-Wilk normality test (*p* > 0.05), paired sample *t*-test was used. For unpaired sample data not satisfying the normality test (*p* < 0.05), the Mann–Whitney *U* test (also named Wilcoxon rank sum test) was used.

### 2.3. Procurement of Clinical Information of TCGA-COADREAD Data Set

The expression levels of GCG mRNA and clinicopathological information of TCGA-COADREAD data set were obtained. The categorical variables included T stage (T1/T2/T3/T4), N stage (N0/N1/N2), M stage (M0/M1), pathologic stage (stages I/II/III/IV), primary therapy outcome (PD/SD/PR/CR), gender (female/male), race (Asian/Black or African American/White), age (≤65/>65), weight (≤90/>90), height (<170/≥170), BMI (<25/≥25), residual tumor (R0/R1/R2), CEA (carcinoembryonic antigen) level (≤5/>5), perineural invasion (no/yes), lymphatic invasion (no/yes), history of colon polyps (no/yes), colon polyps present (no/yes), neoplasm type (colon adenocarcinoma/rectum adenocarcinoma), OS (overall survival) event (alive/dead), DSS (disease specific survival) event (alive/dead), and PFI (progression free survival) event (alive/dead). If all levels of a certain categorical variable satisfied the conditions of theoretical frequency > 5 and total sample size > 40, chi-square test was applied. However, any level in a certain categorical variable that did not satisfy the condition of theoretical frequency > 5 and total sample size > 40, Fisher's exact test was used. If the data for a certain categorical variable were not normally distributed (*p* < 0.05), the Wilcoxon rank sum test was used. All analyses were performed using R (version 3.6.3). Based on the median value of GCG expression level, COADREAD samples corresponding to each level of a certain categorical variable were divided into two groups: low expression group of GCG gene and high expression group of GCG gene.

### 2.4. The Relationship between Clinical Characteristics and GCG Expression

The association of GCG mRNA expression groups with 18 clinical variables was tested: T stage, N stage, M stage, pathologic stage, primary therapy outcome, gender, race, age, weight, height, BMI, residual tumor, CEA level, perineural invasion, lymphatic invasion, history of colon polyps, colon polyps present, and neoplasm type. The relationship between GCG expression and clinical features was also investigated by using binary logistic regression. Logistic regression is a statistical technique used to predict the relationship between predictors (independent variables, herein, the GCG gene expression group) and a predicted variable (dependent variable, herein, characteristics). The independent variable categories included low expression of GCG and high expression of GCG, where the low expression group was used as the reference level. The dependent variables were also grouped into two categories each, T stage (T3 and T4 vs. T1 and T2), N stage (N1 and N2 vs. N0), M stage (M1 vs. M0), pathologic stage (Stage III and Stage IV vs. Stage I and Stage II), primary therapy outcome (PD and SD vs. PR and CR), gender (male vs. female), race (Asian and Black or African American vs. White), age (>65 vs. ≤65), weight (>90 vs. ≤90), height (≥170 vs. <170), BMI (≥25 vs. <25), residual tumor (R1 and R2 vs. R0), CEA level (>5 vs. ≤5), perineural invasion (yes vs. no), lymphatic invasion (yes vs. no), history of colon polyps (yes vs. no), colon polyps present (yes vs. no), and neoplasm type (rectum adenocarcinoma vs. colon adenocarcinoma). Among these characteristics, the latter ones were considered as the reference level.

### 2.5. Survival Analyses to Investigate the Prognostic Value of GCG in COADREAD

Within the TCGA-COADREAD data, only tumor samples with survival information were used for survival analysis. Kaplan-Meier analysis was used to compare the survival curves of high and low GCG gene expression groups, with *p* value determined by log-rank test. The analysis was performed using the “survival” package (version 3.2-10) in R, and the Kaplan-Meier (KM) plots were visualized using the “survminer” package (version 0.4.9) in R. Cox regression analysis was applied, and prognostic parameters analyzed included overall survival (OS), disease specific survival (PSF), and progress free interval (PFI).

### 2.6. Subgroup Survival Analyses

Subgroup survival analysis was performed to investigate if GCG mRNA overexpression could significantly affect the overall survival outcome of COADREAD tumor cases belonging to specific subgroups based on clinical characteristics including T stage, N stage, M stage, pathologic stage, primary therapy outcome, gender, race, age, weight, height, BMI, residual tumor, CEA level, perineural invasion, lymphatic invasion, history of colon polyps, colon polyps present, and neoplasm type. For the subgroup survival analysis, the prognostic parameter was selected was overall survival (OS). Cox regression analysis was applied using the “survival” package (version 3.2-10) in R, and the Kaplan-Meier (KM) plots were visualized by the “survminer” package (version 0.4.9).

### 2.7. Survival Analysis by Univariate Cox Regression

The association between clinical variables and prognosis was investigated by performing univariate Cox regression analyses. The “coxph” function in the R “survival” package (version 3.2-10) was applied, and the cox regression module was used. Overall survival was selected as the outcome. The clinical variables included in this analysis consisted of T stage, N stage, M stage, pathologic stage, primary therapy outcome, gender, race, age, weight, height, BMI, residual tumor, CEA level, perineural invasion, lymphatic invasion, history of colon polyps, colon polyps present, neoplasm type, and GCG expression. The lower T stage (T1 and T2), N stage (N0), M stage (M0), lower pathologic stage (Stage I and Stage II), primary therapy outcome (PR and CR), gender (female), race (White), age (≤65), weight (≤90), height (<170), BMI (<25), residual tumor (R0), CEA level (≤5), without perineural invasion, without lymphatic invasion, without history of colon polyps, without colon polyps present, neoplasm type of colon adenocarcinoma, and higher expression of GCG mRNA levels were used as reference levels.

### 2.8. Forest Plots

Based on the results (HR, 95% CI, *p* value) obtained by univariate cox regression analysis, a forest plot was plotted by using the “ggplot2” package (version 3.3.3) in R (version 3.6.3). HR (hazard ratio) represents a relative risk of death that compares one instance of a binary feature to the other instance, i.e., reference level. An HR > 1 indicates an increased risk of death, while an HR < 1 represents a decreased risk of death as compared to the reference group.

### 2.9. ROC Curves to Evaluate the Diagnostic Value of GCG mRNA Expression in COADREAD

ROC curve analysis of GCG gene expression data was conducted by using the “pROC” package (version 1.17.0.1) and visualized by “ggplot2” package (version 3.3.3) in R (version 3.6.3). The clinical status (COADREAD tumor vs. healthy control) was used as the predicated outcome parameter. In the ROC curves, the *x*-axis represents the false positive rate (false positive rate (FPR)), and the *y*-axis represents the true positive rate (true positive rate (TPR)). The area under the ROC curve (AUC) can be interpreted as that greater than 0.9 having high accuracy, 0.7-0.9 indicating moderate accuracy, 0.5-0.7 indicating low accuracy, and <0.5 indicating a chance result. The AUC and confidence interval (CI) corresponding to the all predicted outcomes were listed, and ROC curves of the predicted outcomes with AUC of greater than 0.7 were presented graphically.

### 2.10. Identification of the Significantly Correlated Genes of GCG in COADREAD

The correlation analysis for the single gene-GCG was performed using the “stat” package (version 3.6.3) in R program (version 3.6.3). Pearson's correlation test, as a parameter correlation test, can measure a linear relationship between two groups and was applied. After performing such analysis, only protein coding genes were retained. The co_pearson value is the Pearson correlation coefficient “r,” where a value of greater than 0.7 is considered a strong correlation, a value between 0.5 and 0.7 indicates a moderate correlation, while a value of less than 0.4 is considered a weak or no correlation. Genes with ∣cor_pearson | >0.4 and p_pearson < 0.001 were defined as significantly correlated genes. A positive value of cor_pearson indicates the positive correlation between GCG and a certain gene, while a negative value indicates a negative correlation of GCG with a certain gene.

### 2.11. Heatmap Plotting of GCG-Top 20 Positively and Negatively Correlated Genes

After defining the significantly correlated genes of GCG, the top 20 genes ranked by descending order of the cor_pearson value were obtained and regarded as the top 20 positively correlated genes of GCG, while the top 20 genes list ranked by the ascending order of the cor_pearson value were obtained and regarded as the top 20 negatively correlated genes of GCG. A heatmap was plotted to show the expression pattern of these 40 correlated genes in COADREAD samples. The heatmap was visualized by using the ggplot2 (version 3.3.3) in R program (version 3.6.3).

### 2.12. The Functional Enrichment Analysis of Significantly Correlated Genes of GCG

The top 100 positively correlated and top 100 negatively correlated genes of GCG were used for functional enrichment analysis to identify significantly enriched functional terms among GCG-correlated genes. The gene names were converted to the Entrez ID by using the “http://org.Hs.eg.db” package (version 3.10.0) in R (version 3.6.3). Functional enrichment analysis was performed using the “clusterProfiler” package (version 3.14.3) in R program (version 3.6.3) with Homo sapiens as the selected species. The Benjamini and Hochberg (BH) method was used for calculating the adjusted *p* values. GO terms, including BP (biological process), CC (cellular component), and MF (molecular function), and KEGG pathways significantly enriched by the correlated genes were identified, setting a threshold of p.adj < 0.05 and *q* value < 0.2. If there were more than 30 terms which were significantly enriched at this threshold setting, then only the top 30 terms ranked by the ascending order of the *p* value were obtained to plot a bubble chart; otherwise, all of the terms were used. The bubble charts were plotted to visualize the enrichment results using the “ggplot2” package (version 3.3.3) in R program (version 3.6.3).

### 2.13. Gene Set Enrichment Analysis (GSEA)

The differentially expressed genes (DEGs), which were significantly dysregulated between COADREAD samples and healthy control samples, were identified by using “DESeq2” (version 1.26.0) in R (version 3.6.3) using the TCGA-COADREAD data set. For the differential expression analysis, the experimental group was established as clinical status-tumor samples, while the reference group was established as clinical status-healthy control samples. The GCG-correlated genes with an absolute value of cor_pearson (r) > 0.1 and *p* value < 0.05 were obtained and used for the GSEA analysis. The log_2_FC (fold change) values of these GCG-correlated genes were obtained and used for the gene set enrichment analysis (GSEA). GSEA analysis was performed using the “clusterProfiler” package (version 3.14.3) in R (version 3.6.3). The pathways were obtained using three databases including KEGG pathway database, WikiPathways (WP) database, and Reactome (REAC) database. The referenced gene set was c2.cp.v7.2.symbols.gmt (curated) in the MSigDB Collections (URL: https://www.gsea-msigdb.org/gsea/msigdb/collections.jsp#C2). The functional terms satisfying the condition of p.adjust < 0.05, false discovery rate (FDR) (also named *q*-val) <0.25, and ∣NES | >1 were considered as significantly enriched terms.

### 2.14. Construction of Gene-Gene Interaction (GGI) and Protein-Protein Interaction (PPI) Network

The GeneMANIA webserver (URL: http://genemania.org) was used for constructing the GCG-based gene-gene interaction network (GGI). GCG gene was used as the input, and top functions with the smallest FDR values were selected for depiction in the network. The network consisted of GCG and its 20 correlated genes. The GGI network was constructed by the automatically selected weighting method. In addition, a protein-protein interaction (PPI) network of GCG coexpressed genes was constructed using a STRING (http://string-db.org) database (version 11.5). The advanced setting was defined as follows: (1) network type: full STRING network, (2) required score: high confidence (0.700), and (3) size cutoff: no more than 20 interactors.

### 2.15. The Correlation between GCG Expression and Immune Cells in COADREAD

The correlation between the GCG gene and immune cells in COADREAD tumor samples was assessed using the Pearson's correlation test. This analysis was performed using the “GSVA” package (version 1.34.0) in R (version 3.6.3). The ssGSEA algorithm, a built-in algorithm in the GSVA package, was used for the statistical analysis. The analyzed 24 tumor immune infiltration cells (TIICs) consisted of aDC (activated DC), B cells, CD8 T cells, cytotoxic cells, DC, eosinophils, iDC (immature DC), macrophages, mast cells, neutrophils, NK CD56bright cells, NK CD56dim cells, NK cells, pDC (plasmacytoid DC), T cells, T helper cells, Tcm (T central memory), Tem (T effector memory), Tfh (T follicular helper), Tgd (T gamma delta), Th1 cells, Th17 cells, Th2 cells, and Treg cells. Lollipop plots were used for illustrating the correlation between GCG expression and 24 TIICs in COADREAD samples. For specific types of immune cells with statistical significance, scatter plots were drawn to depict the correlation between GCG expression and the specific type of cell in COADREAD samples. To further validate the relationship between TAOK1 and diverse immune-infiltrating cells, the associations between GCG and immune marker sets of immune cells were also explored.

## 3. Results

### 3.1. Dysregulation of GCG in Pan-Cancer and COADREAD Data

**Figure 1:**
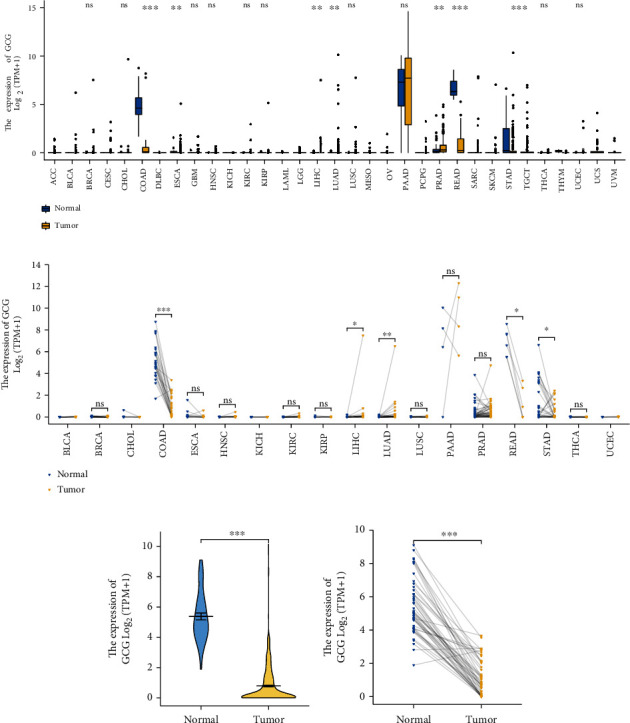
The expression pattern of GCG gene in pan-cancer and COADREAD samples, respectively, using paired and unpaired sample analysis. (a) The expression pattern of GCG gene in pan-cancer using unpaired sample analysis. (b) The expression pattern of GCG gene in pan-cancer using paired sample analysis. (c) The expression pattern of GCG gene in COADREAD using unpaired sample analysis. (d) The expression pattern of GCG gene in COADREAD using paired sample analysis.

The transcription level of GCG gene in pan-cancer was evaluated by analyzing TCGA RNA-seq data and shown in Figures 1(a) and 1(b). Obviously seen from Figures 1(a) and 1(b), GCG mRNA expression was remarkably lower in several cancers (e.g., COAD, READ, and STAD), compared with healthy control samples, while GCG mRNA expression was significantly higher in two other cancers (e.g., LIHC and LUAD), compared with healthy control samples. The transcription level of GCG gene in COADREAD cancer was evaluated by analyzing TCGA RNA-seq data and shown in Figures 1(c) and 1(d). Similar to the results of pan-cancer analysis, Figures 1(c) and 1(d) show that the GCG gene was significantly downregulated in COADREAD samples compared with healthy control oral samples.  Figure 1(c) uses Mann–Whitney *U* test based on 647 COADREAD tumor samples and 51 healthy control samples, and the expression of GCG was lower in COADREAD samples than that in healthy control samples, and the median difference between the two groups was -4.589 (-4.842–-4.133), with statistically significant differences (*p* < 0.001).  Figure 1(d) uses paired sample *t*-test based on 50 COADREAD tumor samples and their 50 paired healthy control samples and shows that the expression of GCG in COADREAD tumor samples was lower than that in healthy control samples, and the median difference between the two groups was -4.273(-4.797–-3.748), with statistically significant differences (*t* = −16.367, *p* < 0.001).

### 3.2. Clinical Characteristics of the TCGA-COADREAD Patients

The clinical and gene expression data of 698 TCGA-COADREAD tumor samples were downloaded from TCGA database (Table S1). Observed from Table S1, only three clinical variables (i.e., race, history of colon polyps, and OS events) were statistically significantly related to the expression of GCG gene (*p* value < 0.05); however, the relationship between any of the other clinical variables and GCG expression was not found to be statistically significant (*p* > 0.05).

### 3.3. Clinical Variables Significantly Correlated with GCG Gene Expression

Table 1 presents the results of logistic analysis and shows the association between GCG gene expression and clinical features of patients with COADREAD. Each row in the table represents a binary logistics regression model. The independent variable is the gene-GCG, and the low expression group of GCG is used as the reference level; the dependent variable is the clinical characteristic. The mRNA expression of GCG was significantly associated with history of colon polyps (yes vs. no: OR = 1.707, 95% confidence interval (CI) = 1.190-2.459, *p* = 0.004); however, the mRNA expression of GCG was not found to be significantly associated with the other clinical variables except for the variable “history of colon polyps.”

### 3.4. Survival Analyses for GCG in Pan-Cancer and COADREAD Data

**Figure 2:**
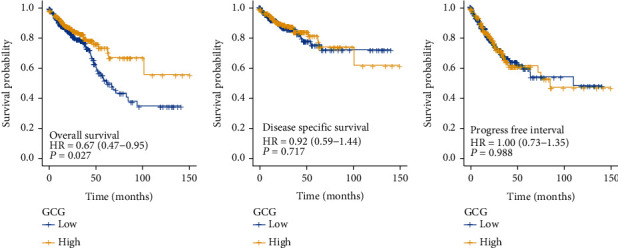
The prognostic value of GCG in COADREAD for three prognostic parameters: overall survival (a), disease specific survival (b), and progress free interval (c).

Figure 2 shows that the difference of overall survival time distribution was statistically significant between the low and high expression groups of GCG (*p* = 0.027), indicating that the high expression group of GCG was associated with better overall survival outcome. There was no statistical difference in disease specific survival time distribution (*p* = 0.717) and progress free interval time distribution between two groups (*p* = 0.988) between the two groups.

### 3.5. Results of Subgroup Survival Analyses

**Figure 3:**
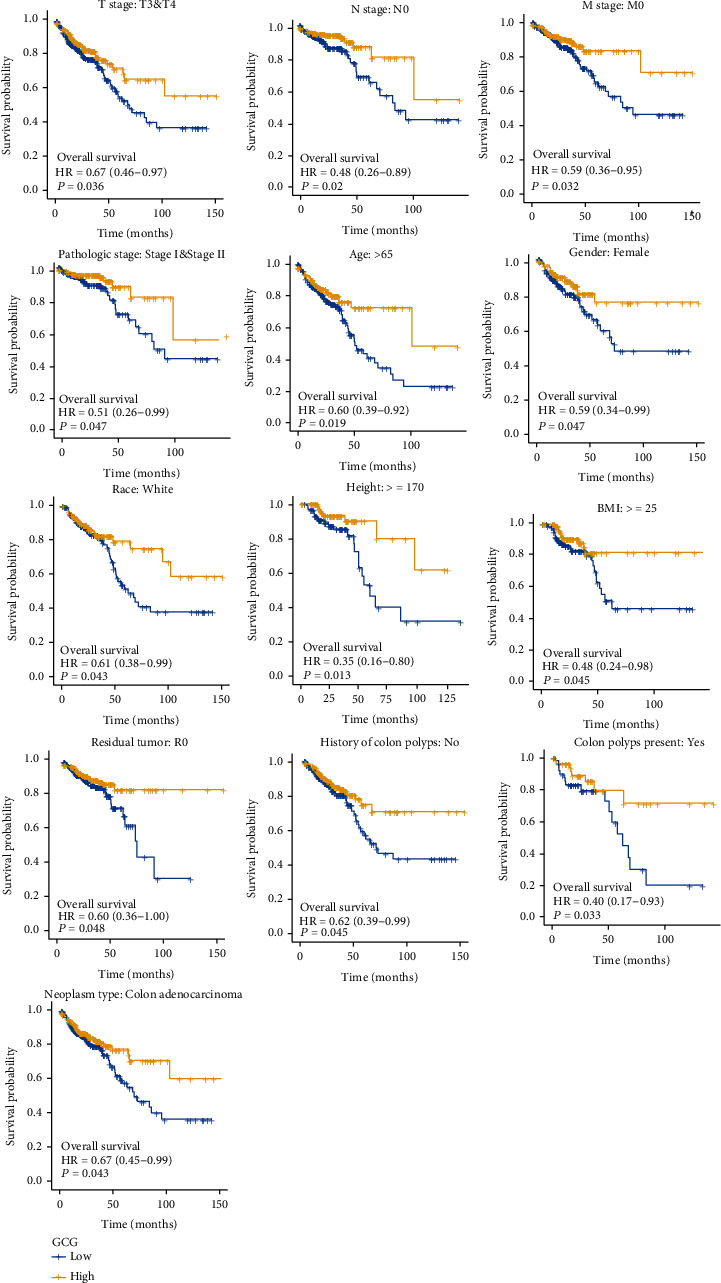
The subgroup survival analysis shows the association of GCG mRNA overexpression with overall survival outcome of COADREAD in specific subgroups based on clinical characteristics including higher T stages; N stage, N0; M stage, M0; lower pathologic stage, Stage I and Stage II; age > 65; gender, female; race, White; height ≥ 170; BMI ≥ 25; residual tumor, R0; without history of colon polyps; colon polyps present, yes; and neoplasm type, colon adenocarcinoma.

Subgroup survival analysis indicated that GCG mRNA overexpression significantly affected the overall survival outcome of COADREAD tumor cases belonging to the subgroups of higher T stages, T3 and T4 (*p* = 0.036); N stage, N0 (*p* = 0.02); M stage, M0 (*p* = 0.032); lower pathologic stage, Stage I and Stage II (*p* = 0.047); age of more than 65 (*p* = 0.019); gender, female (*p* = 0.047); race, White (*p* = 0.043); height ≥ 170 (*p* = 0.013); BMI ≥ 25 (*p* = 0.045); residual tumor, R0 (*p* = 0.048); without history of colon polyps (*p* = 0.045); colon polyps present, yes (*p* = 0.033); and neoplasm type, colon adenocarcinoma (*p* = 0.043), respectively (Figure 3).

### 3.6. Forest Plots Depicting the Results of the Univariate Cox Regression Analyses

**Figure 4:**
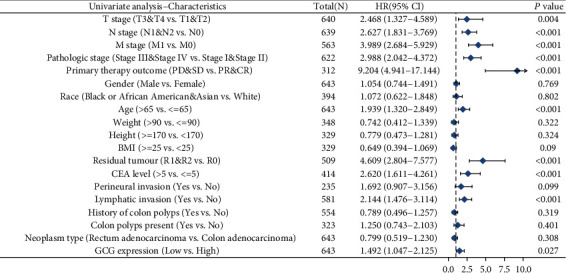
The forest plots showing the univariate regression analysis results of GCG and clinicopathologic parameters with overall survival (OS) in COADREAD patients.

The univariate analysis results showed that ten variables (T stage, N stage, M stage, pathologic stage, primary therapy outcome, age, residual tumor, CEA level, lymphatic invasion, and GCG expression) were statistically significantly related to the overall survival of COADREAD patients.  Figure 4 shows the results of univariate Cox regression analysis, indicating that several factors (e.g., higher T stage (T3 and T4), higher N stage (N1 and N2), higher M stage (M0), higher pathologic stage (Stage III and Stage IV), primary therapy outcome (PD and SD), age, residual tumor (R1 and R2), CEA level (>5), lymphatic invasion (yes), and low level of GCG expression) were negative predictors for overall survival outcome in COADREAD patients. The univariate Cox regression analysis results showed that the GCG expression is an independent prognostic factor correlated with overall survival in COADREAD patients.

### 3.7. Diagnostic Value of GCG mRNA Expression in COADREAD

**Figure 5:**
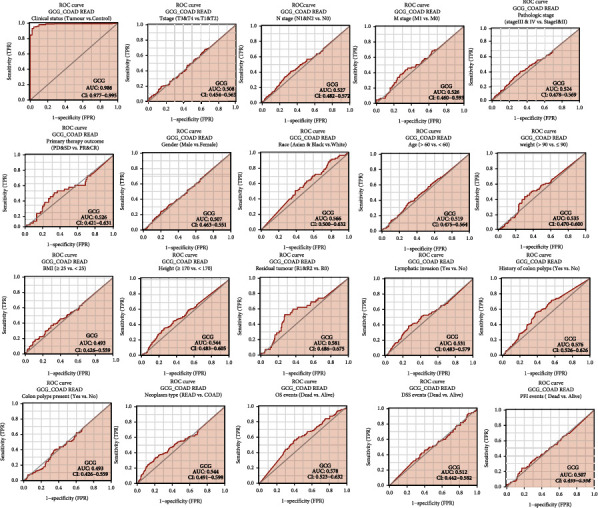
ROC curve analysis to evaluate the diagnostic accuracy of GCG expression for distinguishing various clinicopathological variables of COADREAD.

The diagnostic value of GCG mRNA expression by ROC curve was evaluated. In general, AUC values are interpreted as follows: 0.5-0.6 (failed), 0.6-0.7 (worthless), 0.7-0.8 (poor), 0.8-0.9 (good), and > 0.9 (excellent).  Figure 5 shows that the predictive ability of GCG gene expression has high accuracy in predicting the clinical variables of clinical status (COADREAD tumor versus. Healthy control) (AUC = 0.986, CI = 0.977-0.995). However, the GCG gene expression has low accuracy in predicting the other clinicopathological variables (AUC < 0.6).

### 3.8. Heatmap Showing the Expression Pattern of Top 20 Correlated Genes of GCG in COADREAD

**Figure 6:**
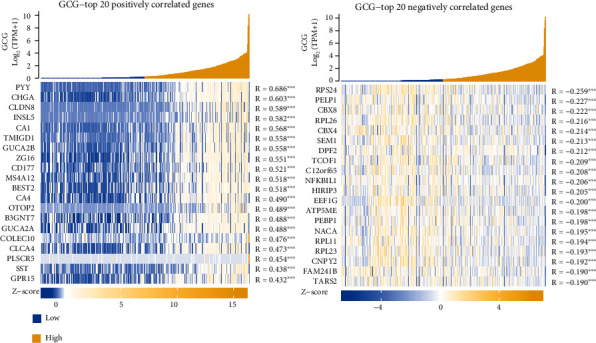
Heatmap showing the expression pattern of the top 20 GCG positively and negatively correlated genes in COADREAD samples.

Among the GCG-significantly correlated genes, the top 20 positively and top 20 negatively correlated genes were obtained. The top 20 positively correlated genes of GCG were found to be PYY, CHGA, CLDN8, INSL5, CA1, TMIGD1, GUCA2B, ZG16, CD177, MS4A12, BEST2, CA4, OTOP2, B3GNT7, GUCA2A, COLEC10, CLCA4, PLSCR5, SST, and GPR15. The top 20 negatively correlated genes of GCG were found to be RPS24, PELP1, CBX8, RPL26, CBX4, SEM1, DPF2, TCOF1, C12orf65, NFKBIL1, HIRIP3, EEF1G, ATP5ME, PEBP1, NACA, RPL11, RPL23, CNPY2, FAM241B, and TARS2.  Figure 6 depicts a heatmap of the expression pattern of the 40 top GCG-correlated genes in COADREAD samples.

### 3.9. Biological Functions of the Significantly Correlated Genes of GCG

**Figure 7:**
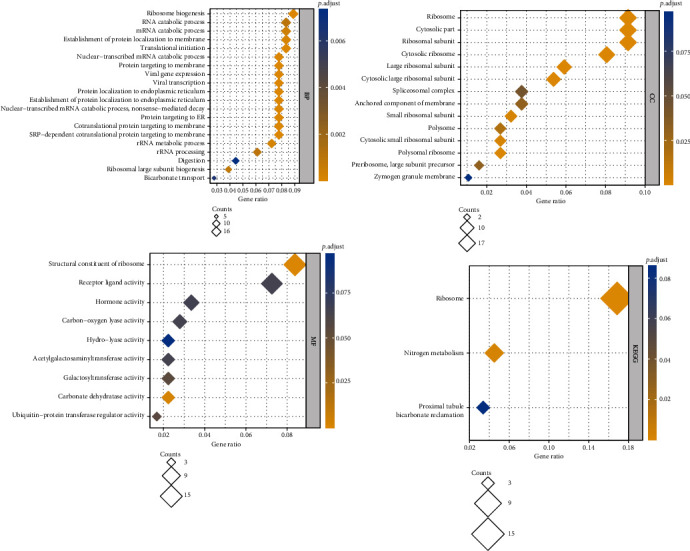
The functional enrichment analysis results of the top 100 positively and top 100 negatively correlated genes of GCG, in terms of GO terms—BP (biological process), CC (cellular component), MF (molecular function), and KEGG pathways.

Figure 7 depicts bubble charts summarizing enrichment results for three GO terms and KEGG pathways enriched by top 100 positively and top 100 negatively correlated genes. GCG-correlated genes were mainly enriched in endoplasmic reticulum- (ER-) related biological processes including protein localization to ER, establishment of protein localization to ER, and protein targeting to ER; ribosome-related cellular components including ribosome, ribosomal subunit, cytosolic ribosome, large ribosomal subunit, small ribosomal subunit, and polysomal ribosome; and molecular functions including transferase activity-related molecular functions, acetrylgalactosaminyl-transferase activity, galactosyl-transferase activity, and ubiquitin-protein transferase regulator activity. Only three KEGG pathways were enriched including ribosome, nitrogen metabolism, and proximal tubule bicarbonate reclamation.

### 3.10. Results of GSEA

**Figure 8:**
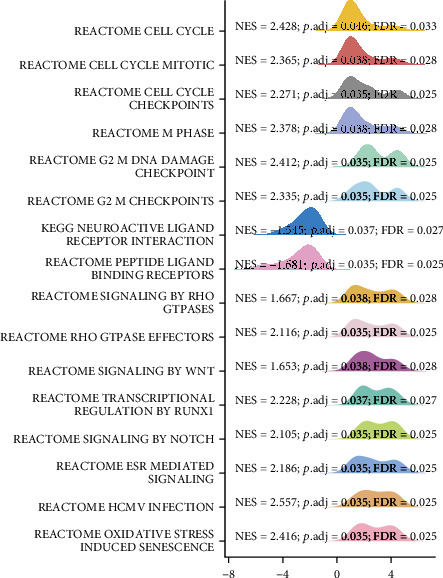
The mountain map showing the GSEA results.

The results of GSEA visualized in Figure 8 show that GCG-significantly correlated genes were mainly enriched in several signaling pathways including cell cycle-related pathways (reactome cell cycle, reactome cell cycle mitotic, reactome cell cycle checkpoints, reactome M phase, Reactome G2 M DNA damage checkpoint, and Reactome G2 M checkpoints), neuropeptide ligand receptor interaction, RHO GTPases signaling, WNT signaling, RUNX1 signaling, NOTCH signaling, ESR signaling, HCMV infection, and oxidative stress-related signaling.

### 3.11. GGI and PPI Network Plotting

**Figure 9:**
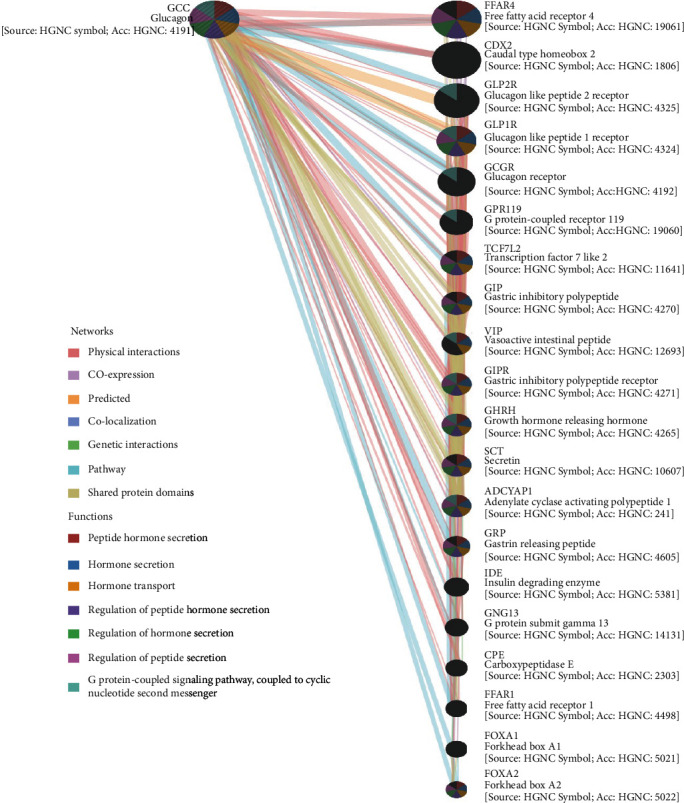
The gene-gene interaction networks constructed by GCG and its 20 coexpressed genes, which was plotted by using GeneMania webserver.

**Figure 10:**
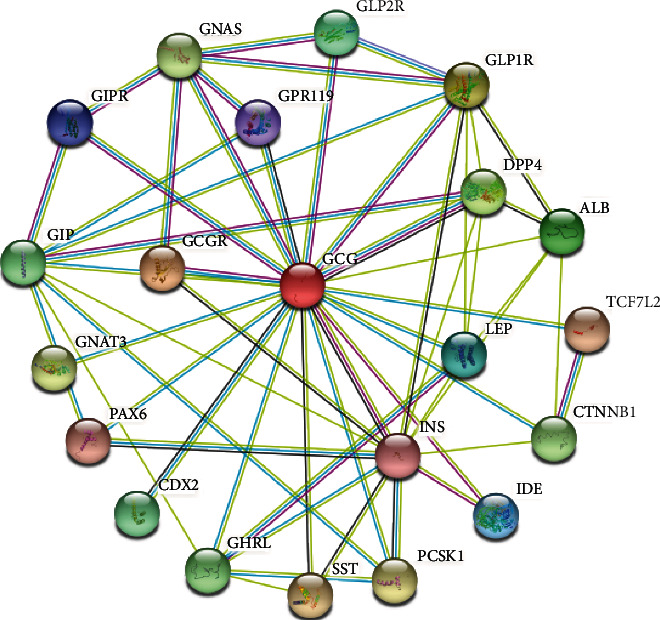
The protein-protein interaction networks constructed by GCG and its 20 interacted genes, which was plotted by using STRING web tool.

As shown in Figure 9, the GCG-based GGI network consisted of the GCG gene and its 20 potentially frequently interacting genes. The 20 correlated genes of GCG were shown as FFAR4 (free fatty acid receptor 4), CDX2 (caudal type homeobox 2), GLP2R (glucagon-like peptide 2 receptor), GLP1R (glucagon-like peptide 1 receptor), GCGR (glucagon receptor), GPR119 (G protein-coupled receptor 119), TCF7L2 (transcription factor 7 like 2), GIP (gastric inhibitory polypeptide), VIP (vasoactive intestinal peptide), GIPR (gastric inhibitory polypeptide receptor), GHRH (growth hormone releasing hormone), SCT (secretin), ADCYAP1 (adenylate cyclase activating polypeptide 1), GRP (gastrin releasing peptide), IDE (insulin degrading enzyme), GNG13 (G protein subunit gamma 13), CPE (carboxypeptidase E), FFAR1 (free fatty acid receptor 1), FOXA1 (forkhead box A1), and FOXA2 (forkhead box A2). The functional analysis of the network described the roles of the 21 genes in terms of biological functions and included peptide hormone secretion, hormone secretion, hormone transport, regulation of peptide hormone secretion, regulation of hormone regulation, regulation of peptide secretion, and G protein-coupled receptor signaling pathway. As shown in Figure 10, GCG coexpressed genes constituted PPI network. GCG was found to interact with genes including GCGR (glucagon receptor), GLP1R (glucagon-like peptide 1 receptor), GLP2R (glucagon-like peptide 2 receptor), GIP (gastric inhibitory polypeptide), GIPR (gastric inhibitory polypeptide receptor), and GPR119 (G protein-coupled receptor 119).

### 3.12. Correlation between GCG Expression and Immune Cells in COADREAD

**Figure 11:**
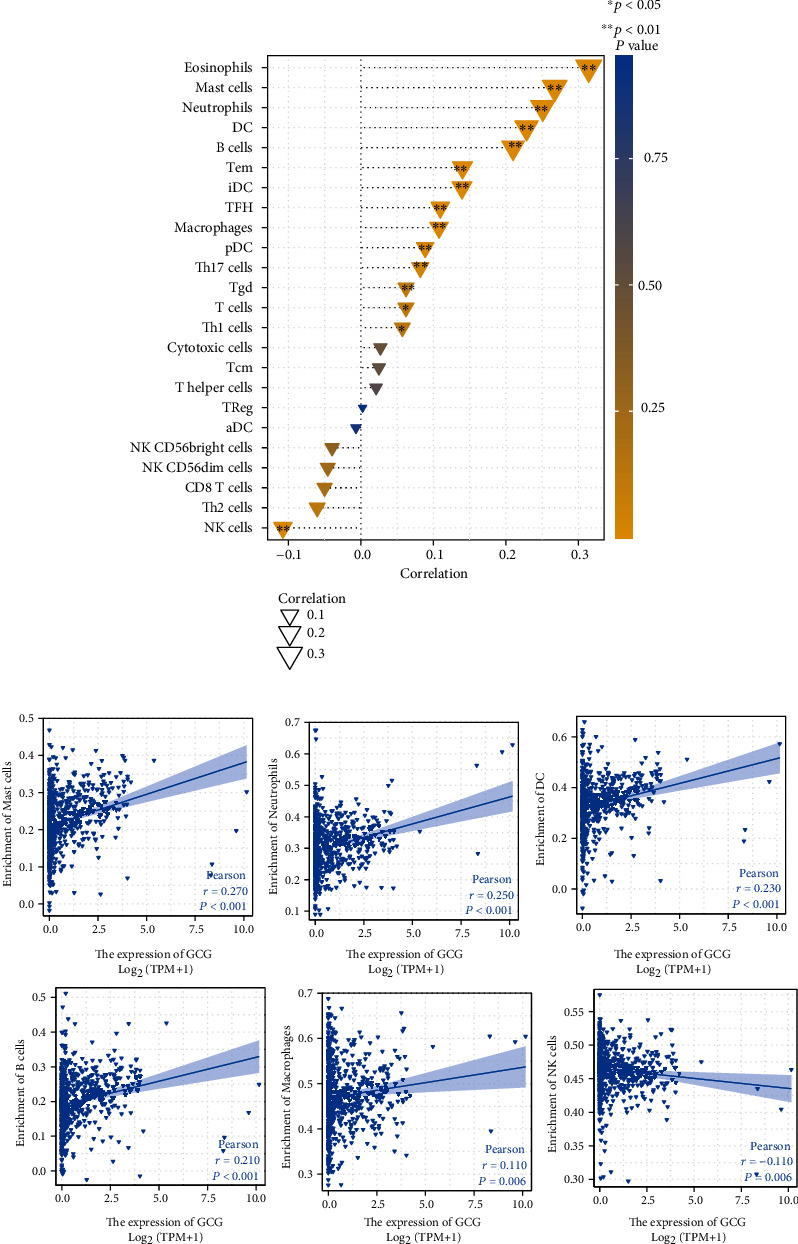
Correlation between GCG expression and tumor immune infiltrating cells. (a) Lollipop plot showing the correlation between GCG expression and 24 TIICs in COADREAD. (b) Scatter plots showing significant positive correlation between GCG expression and 5 types of immune cells (e.g., mast cells, neutrophils, dendritic cells, B cells, and macrophages) and the negative correlation between GCG expression and NK cells.

Figure 11 shows that GCG was significantly positively correlated with several TIICs, including Th17 cells, pDC, macrophages, TFH cells, iDC, Tem, B cells, dendritic cells, neutrophils, mast cells, and eosinophils and significantly negatively correlated with only NK cells. Scatter plots were used to depict the association between GCG expression and several representative cells including mast cells (*r* = 0.270, *p* < 0.001), neutrophils (*r* = 0.250, *p* < 0.001), dendritic cells (*r* = 0.230, *p* < 0.001), B cells (*r* = 0.210, *p* < 0.001), macrophages (*r* = 0.110, *p* = 0.006), and NK cells (*r* = −0.11, *p* = 0.006). Table S2 shows the correlation between GCG expression and surface markers of immune cells. As shown in Table S2, the expression levels of 3 markers of T cells (CD3D, CD3E, CD2), 2 markers of B cells (CD19, CD79A), 2 markers of monocytes (CD86, CSF1R), 3 markers of M2 macrophage (CD163, VSIG4, MS4A4A), and 2 markers of Th17 cells (STAT3, IL17A) have markedly positive correlations with GCG expression in COADREAD.

## 4. Discussion

The preliminary TCGA dataset analysis indicated that GCG expression was downregulated in multiple cancers including COADREAD, although it was upregulated in liver hepatocellular carcinoma and lung adenocarcinoma. Additionally, GCG expression showed very high diagnostic value (AUC = 0.98) for distinguishing COADREAD from controls. GCG, a 29 amino acid long peptide, is produced by alpha cells of pancreas and extrapancreatically from gut epithelial endocrine cells [14] and has multiple functions other that increasing hepatic glucose output including effects on satiety, energy homeostasis, gastrointestinal motility, and renal activity [15]. In contrast, some previous work has shown that hyperglucagonemia promotes colon cancer *in vivo*, noting increased GCG receptor expression in colon cancer tissue, and *in vitro* GCG stimulation led to COADREAD cell proliferation [7, 16]. However, the present finding is broadly in agreement with past bioinformatic analysis that has shown low GCG expression in COADREAD and its association with worse survival [17]. Multiple other primary and secondary analyses of transcriptional data have also reported a low expression of GCG in COADREAD tissue [18–21]. COADREAD is more frequent in diabetic patients as compared to nondiabetics [22–24]. The role of GLPs in COADREAD has been controversial. While GLP-1 activation was found to reduce CT26 colon cancer cell growth and survival [12], GLP-2 was shown to promote colon cancer [11, 25].

Tumor development and growth is marked by changes in DNA methylation patterns, which may be utilized as biomarkers, or for molecular subtyping of cancer [26]. Aberrant DNA methylation of downregulated GCG in colon cancer has been earlier reported by Spisák et al. [27], purportedly due to promoter hypermythylation beginning in the early adenoma stage, in agreement with the present findings. Here, we showed that a significant correlation of methylation site-cg21671120[TSS-160] with GCG expression. Among the clinical characteristics, overall survival in COADREAD was significantly lower in the low GCG expression group, in agreement with past research [17]. However, a positive history of colon polyps was significantly higher in the high GCG expression group. Previous research has shown that GLP-1 receptor activation contributed to increased gut mucosal growth and crypt fission by modulating FGF 1 signaling, and its reduction reversed this effect [28]. GLP-2 was earlier purported to have an intestinotrophic effect in crypt cells mediated by other growth factors and peptides [29]. In an earlier study [30], colon adenoma samples showed loss of expression of GLP-2, while higher expression was noted in cancer, which led the authors to suggest that increased GLP-2 expression may be linked to advanced stages of COADREAD. Race was also linked to GCG gene expression as supported by earlier data [31]. Consensus molecular subtypes of colorectal cancer have been proposed [32]. Here, subgroup analysis and Cox regression analysis showed that GCG expression was significantly linked to overall survival outcome in multiple clinical categories. These data may support clinical characterization of COADREAD patient subgroups amenable to GCG-based molecular biomarker or therapeutics. In addition, a high significant hazard ratio for GCG expression was noted as associated with the type of primary tumor response (HR = 9.2, *p* < 0.001), and future studies should analyze relevant functional mechanisms underpinning these differences and investigate the role of GCG-related signaling in tumor responsiveness.

Functions enriched by the topmost GCG-correlated genes in COADREAD included several endoplasmic reticulum- (ER-) related biological processes. The ER controls protein secretion and also the degradation of unfolded or misfolded proteins through ubiquitin-proteasome system mediated proteolysis [33], which is implicated in COADREAD via control of Wnt signaling mediated cell proliferation [34]. ER stress can disrupt optimal protein folding, leading to activation of the unfolded protein response which is closely linked to cancer progression via multiple pathways such as cell adaptation, immunosuppression, and chemoresistance [35]. ER stress has been previously shown to increase GCG activation via ubiquitin-specific peptidase14, altering glucose homeostasis [36]. Furthermore, ER stress has been shown to retard protective GLP-1 secretion in intestinal L cells [37]. Several cell cycle-related pathways and checkpoints were enriched in COADREAD by GCG correlated genes. Different stages of COADREAD are marked by varying patterns of deregulation of cell cycle-related pathways such as activation of cell cycle checkpoints in the early preinvasive lesions subject to DNA replication phase and G1 > S phase transition in invasive lesions [38], and further work is needed to decipher the role of GCG associated signaling pathways in cell cycle alteration at different stages of carcinogenesis. Well-established tumor-associated pathways including WNT signaling, RUNX1 signaling, and NOTCH signaling were associated with GCG expression in COADREAD, suggesting an interplay with deregulated GCG signaling, such as in altered glucose homeostasis of diabetes mellitus which is a known risk factor [22–24].

GGI network analysis indicated the G-protein coupled free fatty acid receptor 4 (FFAR4) as a top GCG interacting gene in COADREAD. FFAR4 mediates long chain fatty acid stimulated GCG secretion from [39]. FFAR4 has been implicated in colon cancer progression by stimulation of cellular function [40]. These data point to a role of altered GCG/FFAR4 signaling in COADREAD. The Caudal-type homeobox transcription factor 2 (CDX2) gene is documented as a prognostic biomarker of colon cancer with high value in mesenchymal (MS4) molecular subgroup [41] and a tumorigenic role by promotion of anchorage-independent cell growth and anoikis resistance [42]. CDX2 interaction with other transcription factors is implicated in tissue-specific GCG expression [43], plausibly indicating the dysregulation of molecular GCG transcriptional machinery in COADREAD.

GCG expression was positively correlated with expansion of several immune inflammatory tumor infiltrating cells including Th17 cells, pDC, macrophages, TFH cells, iDC, Tem, B cells, dendritic cells, neutrophils, mast cells, and eosinophils and negatively linked with NK cells, suggesting that GCG expression is closely implicated in the tumor immunoinflammatory microenvironment. Proinflammatory Th17 cytokine overexpression is a hallmark of early colorectal cancer and is linked disease progression [44, 45]. Further, colorectal cancer patients characterized by Th17 functional gene clusters have clinically poor prognosis whereas those marked by Th1 cluster have improved disease free survival [46]. NK cell and CD8+ cell infiltration in colorectal cancer has been associated with improved survival outcome [47]. The role of GCG signaling in regulation of NK cell-T cell crosstalk in COADREAD warrants further investigation. Overall, these findings point to the complex role of GCG in regulating the tumor immune microenvironment, whereby a hyperinflammatory environment appears to be associated with upregulation of GCG in COADREAD. Further studies are warranted to understand the immunophenotypes and temporal variation associated with GCG signaling in COADREAD. These findings also raise the issue of GCG receptor modulation in context of COADREAD. The use of GLP-1 receptor agonists, which are safe and promising antidiabetic agents, has been hypothesized to possibly confer risk of COADREAD by influencing Wnt signaling [48], although no short-term effects have been reported [49]. While the present analyses highlighted several GCG associated genes, functional pathways and immune infiltrating cells are possible mechanisms linked to COADREAD; these notions are limited by the lack of experimental data in the current study. These preliminary findings warrant functional investigations in order to further unravel the role of GCG associated molecular pathways in COADREAD, which may be ultimately leveraged for clinical translation.

The limitations of the present study must be acknowledged. The primary limitation is the lack of experimental validation data from cellular, animal, or human COADREAD samples, as included earlier in a similar approach to characterize single gene involvement lung adenocarcinoma [50]. The key GCG-correlated genes, signaling pathways, and tumor immune microenvironment identified in the bioinformatics analysis require experimental evidence for validation. The prognostic value of GCG expression must be investigated prospectively in large clinical samples. Paradoxically, GCG expression in COADREAD was found correlated with worse prognosis, but also higher expression was also correlated with a more hyperinflammatory tumor immune microenvironment, suggestive of tumor immunosuppression. This finding plausibly indicates GCG gene expression pattern and its associated immune-mediated mechanisms may bear differing effects during different stages of disease progression and should be investigated in future experimental studies. Furthermore, the potential of GCG modulation, GLP-1 receptor agonists, and immune-checkpoint inhibitor therapy in context of GCG expression of COADREAD can be investigated in a precision medicine approach to identify tumor subtypes and optimal therapies. While the present study performed an array of analyses, additional investigations, which were not performed in the current study, may include that of immunohistochemistry data from the Human Protein Atlas (HPA) [51]. Such investigations may also include the determination of concordance index to discriminate predicted and real values of survival modeling, leveraging databases such as UALCAN and cBioPortal [52].

## 5. Conclusion

In conclusion, comprehensive bioinformatics analysis showed that GCG was significantly downregulated in COADREAD tumor samples and associated with worse prognostic outcomes. Enrichment analyses showed that GCG-correlated genes in COADREAD were enriched in several tumor-influencing signaling pathways including ribosome, nitrogen metabolism, proximal tubule bicarbonate reclamation, cell cycle-related pathways, neuropeptide ligand receptor interaction, RHO GTPases signaling, WNT signaling, RUNX1 signaling, NOTCH signaling, ESR signaling, HCMV infection, and oxidative stress-related signaling. GCG expression was linked to a proinflammatory immune infiltrating cell milieu including Th17 cells, pDC, macrophages, TFH cells, iDC, Tem, B cells, dendritic cells, neutrophils, mast cells, and eosinophils and negatively associated with NK cells. Further functional studies are warranted to investigate the effects and role of dysregulated GCG signaling in COADREAD.

## Figures and Tables

**Table 1:** Logistic regression analysis results showing the association between GCG expression and clinical characteristics.

Characteristics (reference level)	Total (*N*)	Odds ratio (OR)	*p* value
T stage (T3&T4 vs. T1&T2)	641	0.993 (0.676-1.458)	0.970
N stage (N1&N2 vs. N0)	640	1.153 (0.843-1.579)	0.373
M stage (M1 vs. M0)	564	1.013 (0.644-1.596)	0.956
Pathologic stage (Stage III&Stage IV vs. Stage I&Stage II)	623	1.129 (0.823-1.550)	0.452
Primary therapy outcome (PD&SD vs. PR&CR)	312	0.991 (0.502-1.983)	0.980
Gender (male vs. female)	644	1.013 (0.743-1.380)	0.937
Race (Asian and Black or African American vs. White)	394	0.628 (0.368-1.049)	0.081
Age (>65 vs. ≤65)	644	1.135 (0.831-1.552)	0.426
Weight (>90 vs. ≤90)	348	1.519 (0.953-2.422)	0.078
Height (≥170 vs. <170)	329	1.384 (0.888-2.165)	0.152
BMI (≥25 vs. <25)	329	1.189 (0.741-1.924)	0.477
Residual tumor (R1&R2 vs. R0)	510	1.479 (0.781-2.884)	0.237
CEA level (>5 vs. ≤5)	415	1.025 (0.688-1.527)	0.905
Lymphatic invasion (yes vs. no)	582	1.168 (0.838-1.629)	0.360
Perineural invasion (yes vs. no)	235	1.233 (0.677-2.231)	0.491
History of colon polyps (yes vs. no)	555	1.707 (1.190-2.459)	0.004
Colon polyps present (yes vs. no)	323	1.077 (0.665-1.737)	0.762
Neoplasm type (rectum adenocarcinoma vs. colon adenocarcinoma)	644	1.297 (0.911-1.852)	0.150

## Data Availability

The data analyzed in the present study are publicly available.

## Supplementary Materials

Supplementary Materials Table S1: the clinical characteristics of the TCGA-COADREAD patients depending on the expression level of GCG gene. Table S2: correlation analysis between GCG and surface biomarkers of immune cells.
