# Supplementary material for: The Regulatory Role of Neuropeptide Gene Glucagon in Colorectal Cancer: A Comprehensive Bioinformatic Analysis
Source: Dis Markers. 2022 Mar 18;2022:4262600. doi: 10.1155/2022/4262600 (PMC8956438; doi:10.1155/2022/4262600)
Supplement: Supplementary Materials — Table S1: the clinical characteristics of the TCGA-COADREAD patients depending on the expression level of GCG gene. Table S2: correlation analysis between GCG and surface biomarkers of immune cells. [file 4262600.f1.docx]

**Supplementary Table S1.** The clinical characteristics of the TCGA-COADREAD patients depending on the expression level of GCG gene.

| **Characteristic** | **Low expression of GCG** | **High expression of GCG** | **P value** | **Chi sq. statistic** | **method** |
| --- | --- | --- | --- | --- | --- |
| n | 322 | 322 |  |  |  |
| **T stage, n (%)** |  |  | 0.459 | 2.59 | Chisq.test |
| T1 | 9 (1.4%) | 11 (1.7%) |  |  |  |
| T2 | 56 (8.7%) | 55 (8.6%) |  |  |  |
| T3 | 211 (32.9%) | 225 (35.1%) |  |  |  |
| T4 | 43 (6.7%) | 31 (4.8%) |  |  |  |
| **N stage, n (%)** |  |  | 0.429 | 1.69 | Chisq.test |
| N0 | 189 (29.5%) | 179 (28%) |  |  |  |
| N1 | 77 (12%) | 76 (11.9%) |  |  |  |
| N2 | 53 (8.3%) | 66 (10.3%) |  |  |  |
| **M stage, n (%)** |  |  | 1.000 | 0 | Chisq.test |
| M0 | 231 (41%) | 244 (43.3%) |  |  |  |
| M1 | 43 (7.6%) | 46 (8.2%) |  |  |  |
| **Pathologic stage, n (%)** |  |  | 0.859 | 0.76 | Chisq.test |
| Stage I | 59 (9.5%) | 52 (8.3%) |  |  |  |
| Stage II | 121 (19.4%) | 117 (18.8%) |  |  |  |
| Stage III | 90 (14.4%) | 94 (15.1%) |  |  |  |
| Stage IV | 43 (6.9%) | 47 (7.5%) |  |  |  |
| **Primary therapy outcome, n (%)** |  |  | 1.000 |  | Fisher.test |
| PD | 15 (4.8%) | 18 (5.8%) |  |  |  |
| SD | 2 (0.6%) | 3 (1%) |  |  |  |
| PR | 7 (2.2%) | 9 (2.9%) |  |  |  |
| CR | 115 (36.9%) | 143 (45.8%) |  |  |  |
| **Gender, n (%)** |  |  | 1.000 | 0 | Chisq.test |
| Female | 151 (23.4%) | 150 (23.3%) |  |  |  |
| Male | 171 (26.6%) | 172 (26.7%) |  |  |  |
| **Race, n (%)** |  |  | 0.046 |  | Fisher.test |
| Asian | 11 (2.8%) | 1 (0.3%) |  |  |  |
| Black or African American | 45 (11.4%) | 24 (6.1%) |  |  |  |
| White | 183 (46.4%) | 130 (33%) |  |  |  |
| **Age, n (%)** |  |  | 0.474 | 0.51 | Chisq.test |
| <=65 | 143 (22.2%) | 133 (20.7%) |  |  |  |
| >65 | 179 (27.8%) | 189 (29.3%) |  |  |  |
| **Weight, n (%)** |  |  | 0.100 | 2.71 | Chisq.test |
| <=90 | 156 (44.8%) | 88 (25.3%) |  |  |  |
| >90 | 56 (16.1%) | 48 (13.8%) |  |  |  |
| **Height, n (%)** |  |  | 0.187 | 1.74 | Chisq.test |
| <170 | 103 (31.3%) | 56 (17%) |  |  |  |
| >=170 | 97 (29.5%) | 73 (22.2%) |  |  |  |
| **BMI, n (%)** |  |  | 0.554 | 0.35 | Chisq.test |
| <25 | 68 (20.7%) | 39 (11.9%) |  |  |  |
| >=25 | 132 (40.1%) | 90 (27.4%) |  |  |  |
| **Residual tumor, n (%)** |  |  | 0.494 |  | Fisher.test |
| R0 | 223 (43.7%) | 245 (48%) |  |  |  |
| R1 | 2 (0.4%) | 4 (0.8%) |  |  |  |
| R2 | 14 (2.7%) | 22 (4.3%) |  |  |  |
| **CEA level, n (%)** |  |  | 0.986 | 0 | Chisq.test |
| <=5 | 127 (30.6%) | 134 (32.3%) |  |  |  |
| >5 | 74 (17.8%) | 80 (19.3%) |  |  |  |
| **Perineural invasion, n (%)** |  |  | 0.591 | 0.29 | Chisq.test |
| No | 108 (46%) | 67 (28.5%) |  |  |  |
| Yes | 34 (14.5%) | 26 (11.1%) |  |  |  |
| **Lymphatic invasion, n (%)** |  |  | 0.406 | 0.69 | Chisq.test |
| No | 178 (30.6%) | 172 (29.6%) |  |  |  |
| Yes | 109 (18.7%) | 123 (21.1%) |  |  |  |
| **History of colon polyps, n (%)** |  |  | 0.005 | 7.91 | Chisq.test |
| No | 198 (35.7%) | 179 (32.3%) |  |  |  |
| Yes | 70 (12.6%) | 108 (19.5%) |  |  |  |
| **Colon polyps present, n (%)** |  |  | 0.857 | 0.03 | Chisq.test |
| No | 133 (41.2%) | 91 (28.2%) |  |  |  |
| Yes | 57 (17.6%) | 42 (13%) |  |  |  |
| **Neoplasm type, n (%)** |  |  | 0.177 | 1.83 | Chisq.test |
| Colon adenocarcinoma | 247 (38.4%) | 231 (35.9%) |  |  |  |
| Rectum adenocarcinoma | 75 (11.6%) | 91 (14.1%) |  |  |  |
| **OS event, n (%)** |  |  | 0.010 | 6.55 | Chisq.test |
| Alive | 244 (37.9%) | 271 (42.1%) |  |  |  |
| Dead | 78 (12.1%) | 51 (7.9%) |  |  |  |
| **DSS event, n (%)** |  |  | 0.831 | 0.05 | Chisq.test |
| Alive | 261 (42%) | 283 (45.5%) |  |  |  |
| Dead | 39 (6.3%) | 39 (6.3%) |  |  |  |
| **PFI event, n (%)** |  |  | 0.857 | 0.03 | Chisq.test |
| Alive | 241 (37.4%) | 238 (37%) |  |  |  |
| Dead | 81 (12.6%) | 84 (13%) |  |  |  |
| **Age, meidan (IQR)** | 68 (57, 76) | 68 (59, 75.75) | 0.478 | 50166 | Wilcoxon |

**Table S2.** Correlation analysis between GCG and surface biomarkers of immune cells.

| **Description** | **Gene Markers** | **correlation pearson** | **p value** |
| --- | --- | --- | --- |
| **CD8+ T cell** | **CD8A** | 0.076 | 0.053 |
|  | **CD8B** | -0.048 | 0.225 |
| **T cell (general)** | **CD3D** | 0.101 | 0.011 |
|  | **CD3E** | 0.088 | 0.025 |
|  | **CD2** | 0.101 | 0.01 |
| **B cell** | **CD19** | 0.207 | <0.001 |
|  | **CD79A** | 0.238 | <0.001 |
| **Monocyte** | **CD86** | 0.131 | <0.001 |
|  | **CSF1R** | 0.177 | <0.001 |
| **TAM** | **CCL2** | 0.19 | <0.001 |
|  | **CD68** | -0.116 | 0.003 |
|  | **IL10** | 0.22 | <0.001 |
| **M1 Macrophage** | **NOS2** | 0.065 | 0.097 |
|  | **IRF5** | -0.003 | 0.94 |
|  | **PTGS2** | 0.15 | <0.001 |
| **M2 Macrophage** | **CD163** | 0.17 | <0.001 |
|  | **VSIG4** | 0.176 | <0.001 |
|  | **MS4A4A** | 0.183 | <0.001 |
| **Neutrophils** | **CEACAM8** | 0.069 | 0.08 |
|  | **ITGAM** | 0.103 | 0.008 |
|  | **CCR7** | 0.189 | <0.001 |
| **Natural killer cell** | **KIR2DL1** | -0.067 | 0.087 |
|  | **KIR2DL3** | -0.021 | 0.595 |
|  | **KIR2DL4** | 0.019 | 0.629 |
|  | **KIR3DL1** | -0.045 | 0.252 |
|  | **KIR3DL2** | 0.007 | 0.861 |
|  | **KIR3DL3** | 0.069 | 0.08 |
|  | **KIR2DS4** | -0.05 | 0.208 |
| **Dendritic cell** | **HLA-DPB1** | 0.091 | 0.02 |
|  | **HLA-DQB1** | 0.036 | 0.358 |
|  | **HLA-DRA** | 0.103 | 0.009 |
|  | **HLA-DPA1** | 0.126 | 0.001 |
|  | **CD1C** | 0.208 | <0.001 |
|  | **NRP1** | 0.083 | 0.036 |
|  | **ITGAX** | 0.152 | <0.001 |
| **Th1** | **TBX21** | 0.013 | 0.746 |
|  | **STAT4** | 0.118 | 0.003 |
|  | **STAT1** | 0.018 | 0.643 |
|  | **IFNG** | -0.008 | 0.835 |
|  | **TNF** | 0.11 | 0.005 |
| **Th2** | **GATA3** | 0.048 | 0.223 |
|  | **STAT6** | -0.07 | 0.076 |
|  | **STAT5A** | -0.087 | 0.028 |
|  | **IL13** | 0.043 | 0.275 |
| **Tfh** | **BCL6** | 0.037 | 0.35 |
|  | **IL21** | 0.014 | 0.731 |
| **Th17** | **STAT3** | 0.107 | 0.007 |
|  | **IL17A** | 0.11 | 0.005 |
| **Treg** | **FOXP3** | 0.077 | 0.049 |
|  | **CCR8** | 0.074 | 0.059 |
|  | **STAT5B** | -0.001 | 0.97 |
|  | **TGFB1** | 0.06 | 0.127 |
| **T cell exhaustion** | **PDCD1** | 0.028 | 0.475 |
|  | **CTLA4** | 0.077 | 0.049 |
|  | **LAG3** | 0.046 | 0.242 |
|  | **HAVCR2** | 0.111 | 0.005 |
|  | **GZMB** | 0.02 | 0.619 |
